# Antidiabetic, hypolipidemic, and antioxidative properties of aqueous and ethanolic extracts of Sage (*Salvia officinalis* L.) against streptozotocin-induced diabetes and oxidative stress in Wistar albino male rats

**DOI:** 10.14202/vetworld.2025.461-474

**Published:** 2025-02-26

**Authors:** Yousef M. Alharbi, Rashed A. Aljalis, Hassan Barakat

**Affiliations:** 1 Department of Medical Biosciences, College of Veterinary Medicine, Qassim University, Buraydah, 51452, Saudi Arabia; 2 Military Industries Corporation, Ministry of Defense, King Khalid Rd, Al-Kharj, 16274, Saudi Arabia; 3 Department of Food Science and Human Nutrition, College of Agriculture and Food, Qassim University, Buraydah, 51452, Saudi Arabia; 4 Department of Food Technology, Faculty of Agriculture, Benha University, Moshtohor, 13736, Egypt

**Keywords:** Antioxidants, diabetes mellitus, hypoglycemic, oxidative stress, *Salvia officinalis*

## Abstract

**Background and Aim:**

Diabetes mellitus (DM) is a chronic disease characterized by insulin insufficiency and hyperglycemia, often leading to complications such as oxidative stress, dyslipidemia, and organs damage. Sage (*Salvia officinalis* L.), a medicinal plant with rich antioxidant and bioactive compounds, has shown promise in managing diabetes and related complications. This study investigates the antidiabetic, hypolipidemic, and antioxidative effects of aqueous (AE) and ethanolic (EE) extracts of *S. officinalis* leaves at doses of 400 and 800 mg/kg body weight in Wister albino male rats with streptozotocin (STZ)-induced type 2 diabetes and oxidative stress.

**Materials and Methods:**

Wistar albino male rats (n = 49) were divided into seven groups: Normal control, diabetes-induced control (STZ), metformin-treated (50 mg/kg/day), and groups treated with AE (400 and 800 mg/kg/day) and EE (400 and 800 mg/kg/day). Parameters assessed included weight gain percentage, random blood glucose (RBG), fasting blood glucose (FBG), lipid profiles, liver and kidney function markers, oxidative stress biomarkers (glutathione [GSH], catalase [CAT], superoxide dismutase [SOD], malonaldehyde [MDA]), and histopathological examination of the pancreas.

**Results:**

AE and EE significantly reduced RBG and FBG and improved weight gain recovery. At 800 mg/kg, AE and EE effectively reduced triglycerides, total cholesterol, low-density lipoproteins cholesterol (LDL-C), and very LDL-C (VLDL-C) while increasing high-density lipoproteins cholesterol more than 400 mg/kg doses or metformin. Liver and kidney functions were restored with high-dose AE and EE showing superior efficacy. Antioxidant biomarkers (GSH, CAT, and SOD) were significantly enhanced, while MDA levels were reduced. Histopathological analysis confirmed restoration of islets of Langerhans and acinar cells to near-normal conditions in treated groups.

**Conclusion:**

The AE and EE of *S. officinalis* demonstrated potent antidiabetic, hypolipidemic, and antioxidative properties, offering significant potential as a natural therapeutic option for managing diabetes and oxidative stress-related complications.

## INTRODUCTION

Diabetes mellitus (DM) is a chronic disease characterized by insulin insufficiency and hyperglycemia. The worldwide prevalence of diabetes was estimated at 9.3% (463 million people) in 2019, rising to 10.2% (578 million) by 2030 and 10.9% (700 million) by 2045, as reported by the International Diabetes Federation [1]. There are two types of DM: Type 1 DM (T1DM) and T2DM. T1DM is a condition of absolute insulin deficiency characterized by islet-directed autoimmunity [2], whereas T2DM is characterized by insulin resistance and a steady reduction in pancreatic β-cell activity. Moreover, it is associated with a higher risk of cardiovascular disease and comorbidities [3]. Various plant extracts have recently been tested for their antioxidant and hypoglycemic activities [4, 5]. Medicinal plants prevent diabetes by inhibiting the small intestine’s α-glucosidase [6], suppressing salivary gland α-amylase [7], enhancing insulin secretion [8], reducing HbA1c and glycated plasma protein [9], increasing glucagon-like peptide-1, and regulating GLUT4 levels [10]. Sage (*Salvia officinalis* L.) is a perennial round shrub belonging to the family of *Labiatae/Lamiaceae* [11]. It has been widely recognized for its antioxidant properties, and the most active components have been identified [12]. Folk medicine treats different disorders, including seizures, ulcers, gout, rheumatism, inflammation, dizziness, tremors, paralysis, diarrhea, and hyperglycemia. Literature indicates that ingesting sage has no adverse effects [11]. It is also used to treat kidney and gallbladder stones, heart illnesses, nerve disorders, headaches, stomachaches, abdominal pain, and other health difficulties. Some cultures use fresh leaves to alleviate hypotension and respiratory issues [13]. In addition, it has anti-inflammatory, antimicrobial, antitumor, and antidiabetic properties. Moreover, it improves cognitive and memory abilities and may prevent or treat Alzheimer’s disease [14]. *S. officinalis* may also alleviate diarrhea and menopausal symptoms [15]. Moreover, *S. officinalis* extract inhibited monoamine neurotransmitter metabolism-related enzymes, indicating the possibility of dopaminergic, serotonergic, and cholinergic actions mediating previously observed improvements [16]. Alharbi *et al*. [17] reported that fermented camel milk containing *S. officinalis* extract protects rats against diabetes and oxidative stress.

The most recognized pathophysiological reasons for *S. officinalis*’ hypoglycemic effectiveness are blocking necessary digestion enzymes, activating and upregulating signaling pathways, and increasing anti-inflammatory and antioxidant actions [18]. Various *S. officinalis*-related treatments inhibit digestive enzymes such as pancreatic α-amylase and intestine α-glucosidase [19–21]. Furthermore, *S. officinalis* compounds such as quercetin, lutein, and kaempferol inhibit glucokinase and glucose 6-phosphatase enzymes. By suppressing these enzymes, gluconeogenesis and insulin sensitivity are increased in hepatocytes [22]. In STZ-induced diabetic rats, *S. officinalis* tea did not affect the effectiveness of Metformin (MET) in lowering glucose production in hepatocytes, indicating that it did not interfere with MET therapy [23]. In addition, lipase activity was inhibited by Koubaa-Ghorbel *et al*. [24]. *S. officinalis* regulates cholesterol excretion, lowers cholesterol production, and inhibits adipocyte lipogenesis [25]. The hypolipidemic and hypoglycemic effects of *S. officinalis* are linked to peroxisome proliferator-activated receptor-gamma activation [26], a nuclear receptor protein that regulates glucose and lipid metabolism and may be used to treat metabolic disorders. *S. officinalis* also increases GLUT4, which regulates glucose homeostasis [27]. Several studies have found that *S. officinalis* enhances the expression of oxidative stress biomarkers [17, 28].

*S. officinalis* has growing evidence of its potential to prevent or treat various health disorders. It can exert different health-promoting activities, such as anti-inflammatory, anti-microbial, anti-tumor, and antidiabetic activities. It has also been shown to promote enhancements in cognitive and memory functions; some studies have reported its efficiency in preventing or improving Alzheimer’s disease [14, 29]. Although lifestyle changes are the first-line treatment for diabetes, medicines are the mainstay. Dietary treatments could significantly reduce diabetic complications and health risks. Thus, functional meals for diabetes management are crucial. Few *in vivo* and in vitro studies have examined *S. officinalis*’ antidiabetic properties [11, 17, 30, 31].

*S. officinalis* is a promising candidate for diabetes management with reduced complications. *S. officinalis* supports the prevention of diabetes and the management of pathophysiological mechanisms. Further research is needed to understand the bioactive components of *S. officinalis* and their antidiabetes efficiency. Therefore, this study investigated the effects of the alcoholic and aqueous (AE) extracts of *S. officinalis* leaves on antidiabetic and antioxidative effects in streptozotocin (STZ)-induced type 2 diabetes and oxidative stress in Wistar albino male rats.

## MATERIALS AND METHODS

### Ethical approval

The study was approved by the Committee of Research Ethics, Deanship of Scientific Research, Qassim University (Approval No. 22-06-03 on Monday, October 10, 2022), SA.

### Study period and location

The study was conducted from January 15 to March 30, 2023, in the animal house of the Department of Food Science and Human Nutrition, College of Agriculture and Food, Qassim University.

### STZ

Experimental T2DM was induced in Wister albino male rats with an injection of STZ (50 mg/kg). STZ was purchased from Sigma–Aldrich, USA. A pharmaceutical-grade formulation of STZ (Alfa Aesar Thermo Fisher Scientific Chemicals, Inc. Co. USA) and MET (Glucophage) was purchased from a local pharmacy (Buraydah, Saudi Arabia).

### *S. officinalis* raw leaves

One kg of air-dried sage (*S. officinalis* L.) was purchased from El Resieny market, Buraydah City, KSA. A plant expert (Dr. Mokded Rabhi, Associate Professor of plant physiology) from Qassim University’s College of Agriculture and Food authenticated the botanical identity of the plant. The leaves were then used to prepare AE and ethanolic (EE) extracts.

### Preparation of AE and EE extracts

#### Preparation of the AE extract

To prepare *S. officinalis* AE extract, 500 g of dried leaves were mechanically crushed and extracted 3 times with 2500 mL of hot water. The filtered extract was concentrated using a rotary evaporator at 40°C, frozen overnight, and freeze-dried for 96 h at 52°C (CHRIST, alpha 1–2 LD plus, Osterode, Germany) under 0.032 mbar [17]. Freeze-dried samples were pulverized with a porcelain mortar to obtain homogenous powder and stored in a dark package at 4 ± 1°C until required.

### Preparation of the EE extract

The *S. officinalis* EE extract was prepared by extracting 500 g with 2500 mL ethanol (70%) 3 times. The filtered extract was concentrated at 40°C using a rotary evaporator, frozen overnight, and freeze-dried for 96 h at 52°C (CHRIST, alpha 1–2 LD plus, Osterode) under 0.032 mbar [17]. Freeze-dried samples were pulverized using a porcelain morsel to prepare homogeneous powder and kept in a dark package at 4 ± 1°C until used.

### Experimental animal design

The animals were rendered diabetic by a single intraperitoneal injection (i.p.) of STZ after the injection of nicotinamide (Alfa Aesar Thermo Fisher Scientific Chemicals, Inc. Co.). Diabetes was induced using the methods described by Shiju *et al*. [32]. STZ was freshly prepared in 0.1 M citrate buffer (pH = 4.5) at a dose of 50 mg/kg; the STZ–injected Wister albino male rats were then given 5 % Glucose solution for 24 h following STZ injection to prevent initial drug-induced hypoglycemic mortality. After 72 h of STZ injection, blood was drawn from the retro-orbital plexus of the rats, and fasting blood sugar was estimated using a glucometer (Accu-Chek, Roche, Germany). Animals with fasting blood glucose (FBG) levels >200–250 mg/dL were included in the treatment. Treatment was started 4 days after the induction of diabetes.

### Animal model

Wister albino male rats weighing 180–200 g (6–8 weeks old) were obtained from the Animal House of the Pharmacy College, King Saud University (Riyadh, Saudi Arabia). The rats were housed in polypropylene cages under standard laboratory conditions (22 ± 3°C, 40–60% humidity, 12-h light/dark cycle) and supplied with a basal diet and water *ad libitum*. The rats were acclimated for 1 week before the study. Forty-nine rats were divided into two groups; the first group (n = 7) was the normal rats (NR) group, and the second group (n = 42) was intended for injection by STZ to produce STZ-induced diabetic rats (DR). The antidiabetic and hypolipidemic properties of AE and EE at 400 and 800 mg/kg were investigated in a designed rat model of six treated groups for 6 weeks. The second group (DR) was divided into G2, the positive group (DR), received a single dose of STZ at 50 mg/kg by i.p. injection; and G3, diabetic rats (DR + Met) received 50 mg MET/kg orally/daily. MET was given to the comparative control group at a dose of 500 mg/kg BW, according to Meng *et al*. [33], which was calculated using the equation presented by Reagan-Shaw *et al*. [34]. G4 (DR + AE400) received 400 mg of *S. officinalis* AE/kg orally/daily; G5 (DR + AE800) received 800 mg of *S. officinalis* AE/kg orally/daily; G6 (DR + EE400) received 400 mg of *S. officinalis* EE/kg orally/daily; and G7 (DR + EE800) received 800 mg of *S. officinalis* EE/kg orally/daily, while, G1 was treated as the negative control group.

Interestingly, *S. officinalis* has abundant evidence of its bioactivity and efficacy; however, the recommended dose for dry *S. officinalis* leaves is 4000–6000 mg daily [35, 36]. In our study, the selected concentrations did not need more toxicity evidence because the examined doses were expected to be lower than recommended [35, 36]. The suggested dose of this study was 400 and 800 mg/kg to be around the used doses before [30, 37]. Kianbakht *et al*. [38] used 150 mg/kg and compared to 500 mg/kg. Consistently, serum lipid levels were enhanced in hyperlipidemic patients taking *S. officinalis* tablets (500 mg/kg) every 8 h daily for 2 months compared with placebo tablets [38]. Furthermore, *S. officinalis* could also inhibit insulin resistance; among euglycemic women diagnosed with polycystic ovary syndrome, consuming *S. officinalis* extract at a dose of 330 mg/day for 8 weeks significantly improved their insulin resistance compared with the placebo group [39].

### Weight measurements

The body weight of each rat was measured weekly throughout the experiment. The cumulative weight gain was calculated according to the following formula:

Cumulative weight gain (g) = Final body weight (g) − Initial body weight (g)

### Blood collection

At the end of the experiment, the experimental rats were anesthetized, and blood samples were collected from the jugular vein. Immediately following collection, the blood tubes were centrifuged (4000 rpm at 10°C for 30 min), and the obtained serum was stored at –18°C until use.

### Determination of FBG

According to Barham and Trinder [40], using the GOD-PAP method, an enzyme colorimetric test kit was used to measure FBG (mg/dL).

### Lipid profile

Total cholesterol (CHO, mg/dL) and triglycerides (TG, mg/dL) were measured using an enzymatic colorimetric test kit following the GPO-PAP protocol [41], and high-density lipoproteins [HDL], mg/dL) were measured with an enzymatic colorimetric direct homogeneous test kit [42]. Low-density lipoprotein (LDL, mg/dL) and very-LDL (VLDL, mg/dL), according to Friedewald *et al*. [43], and Atherogenic index (AI), according to Nwagha *et al*. [44] were mathematically calculated.

### Determination of liver and kidney functions

Liver functions, such as alanine aminotransferase (ALT, U/L), aspartate aminotransferase (AST, U/L), alkaline phosphatase (ALP, U/L), total bilirubin (T. Bili, mg/dL), total protein (T. protein, g/dL), and albumin (g/dL) in blood serum were determined using relevant kits according to the instructions of the manufacturer. Globulin (g/dL) was calculated by subtracting albumin from T. protein concentrations. Using test kits, kidney functions, such as creatinine (mg/dL) and urea (mg/dL) concentrations, were determined according to the manufacturer’s instructions. Blood urea nitrogen (BUN, mg/dL) was calculated by multiplying the urea concentration by 0.47. All biochemical examination kits were purchased from Human Co. (Wiesbaden, Germany) and analyzed using HumaLyzer 4000 (Human Gesellschaft für Biochemica and Diagnostica mbH, Wiesbaden).

### Oxidative stress biomarkers

A glutathione (GSH) colorimetric test kit was used to calculate reduced GSH (g/dL) levels following the protocol reported by Beutler *et al*. [45]. Lipid peroxidation was estimated using a malondialdehyde (MDA, nmol/mL) colorimetric assay kit, according to Ohkawa *et al*. [46]. Superoxide dismutase (SOD, U/L) activity was determined using a SOD-type activity assay kit according to Giannopolitis and Ries [47]. Catalase (CAT, U/L) activity was determined using a CAT activity assay kit according to the Aebi method [48]. All Oxidative stress biomarkers were determined using a blood chemistry analyzer (HumaLyzer 4000, HUMAN Gesellschaft für Biochemica und Diagnostica mbH, Wiesbaden).

### Histopathology

Autopsy samples were collected from the pancreas of rats in different groups and fixed in 10% formalin for 24 h. Washing was then performed under tap water, and serial dilutions of alcohol (methyl, ethyl, and ethyl absolute) were used for drying. The samples were flushed in xylene and embedded in paraffin at 56°C in a hot-air oven for 24 h. Paraffin beeswax tissue blocks were prepared for cutting at 4 μm by sliding microtome. After Microtome sectioning, tissue sections were deparaffinized and immediately stained with hematoxylin and eosin (H&E). The stained sections were diagnosed for histopathological alterations in pancreatic architecture, and their photomicrographs (Zeiss microscope, Germany, equipped with Olympus camera, Malaysia) were taken as described by Banchroft *et al*. [49]. Subsequently, the outcomes of the undefined experimental groups were re-diagnosed by two pathologists to confirm the reproducibility of the results.

### Statistical analysis

Data were analyzed using the Statistical Package for the Social Sciences software (version 22.0 for Windows, IBM, Houston, Texas, USA). All experimental results were expressed as mean ± standard error (SE). The normality of the data was tested using the Shapiro–Wilk test and the homogeneity of variance was assessed using Levene’s test. Statistical comparisons between groups were performed using a one-way analysis of variance followed by Tukey’s *post hoc* test to evaluate pairwise differences [50].

A p < 0.05 was considered statistically significant for all analyses. For non-normally distributed data or when variances were unequal, the Kruskal–Wallis test was applied, followed by Dunn’s *post hoc* test. Graphical data representations were created for clarity using GraphPad Prism (Version 9.0, GraphPad Software, USA).

Correlation analyses were conducted to assess relationships between biochemical parameters and histological findings. All tests were two-tailed, and appropriate statistical methods were chosen based on the specific dataset characteristics.

## RESULTS

### Efficiency of the AE and EE extracts of *S. officinalis* leaves in terms of weight gain

Table 1 shows the effects of AE and EE of *S. officinalis* leaves at 400 and 800 mg/kg and MET at 50 mg/kg on weight gain % in STZ-induced diabetes in rats. STZ injection directly affected rats’ weight during the 1^st^ weeks. However, no weight gain percentage was detected on weeks 2, 4, and 6. The most effective treatment for weight recovery in rats was 800 mg/kg of EE from *S. officinalis* leaves, followed by 800 mg/kg of AE from *S. officinalis* leaves or MET on weeks 4 and 6. Compared with normal rats (G1) or diabetes rats (G2), the AE and EE of *S. officinalis* leaves at 400 mg/kg had the lowest weight growth booster on week 2. After 4 weeks, a remarkable increase was noticed with better improvement when high doses of AE or EE *S. officinalis* were used, demonstrating a practical efficacy in improving weight gain more efficiently than MET at 50 mg/kg, as shown in Table 1. The effect of 800 mg AE or EE of *S. officinalis* leaves Kg^-1^ on weight gain was clearly observed after 6 weeks, confirming the efficiency of high doses, which gave better results than MET in comparable matter (G3).

**Table 1:** Effect of *S. officinalis* AE and EE extracts on weight gain percentage in STZ-induced type 2 diabetes and oxidative stress in experimental rats (mean ± SE), n = 7.

Groups	Weight gain (%)		

	Weak-2	Weak-4	Weak-6
NR	12.04 ± 1.86^a^	20.12 ± 2.45^a^	25.12 ± 2.26^a^
STZ	−11.25 ± 2.94^d^	−13.08 ± 4.11^d^	−9.65 ± 4.24^d^
MET	0.78 ± 2.90^bc^	2.46 ± 2.97^bc^	7.38 ± 2.91^bc^
STZ + AE400	−3.93 ± 7.18^c^	3.16 ± 8.78^bc^	4.58 ± 9.05^bc^
STZ + AE800	−2.81 ± 4.36^cd^	5.11 ± 4.65^bc^	8.71 ± 5.49^bc^
STZ + EE400	−5.10 ± 3.18^cd^	2.13 ± 3.83^cd^	3.94 ± 3.74^cd^
STZ + EE800	3.88 ± 1.71^ab^	12.15 ± 2.63^a^	17.42 ± 4.10^ab^

G1=The negative group, G2=The positive group, G3=Diabetic rats + 50 mg Metformin/kg daily, G4=Diabetic rats + 400 mg of *S. officinalis* AE/kg daily, G4=Diabetic rats + 800 mg of *S. officinalis* AE/kg daily, G6=Diabetic rats + 400 mg of *S. officinalis* EE/kg daily, and G7=Diabetic rats + 800 mg of *S. officinalis* EE/kg daily, ^a,b,c,d^No significant difference (p > 0.05) between any two means within the same column have the same superscripted letters. *S. officinalis*=*Salvia officinalis*, AE=Aqueous, EE=Ethanolic, STZ=Streptozotocin, MET=Metformin, SE=Standard error, NR=Normal rats

### The hypoglycemic efficiency of AE and EE extracts of *S. officinalis* leaves

Table 2 shows the random blood glucose (RBG), and FBG results for AE and EE of *S. officinalis* leaves at 400, 800 mg/kg, and MET at 50 mg/kg on STZ-induced diabetes in rats. Both the AE and EE of *S. officinalis* leaves at 800 mg/kg or MET at 50 mg/kg effectively reduced the RBG more effectively when using 400 mg/kg than when using 800 mg/kg. FBG measurements confirmed that the AE and EE of *S. officinalis* leaves attenuated FBG closely to that of normal rats after 6 weeks of treatment. Interestingly, the EE of *S. officinalis* leaves was significantly better than the AE of *S. officinalis* leaves compared with the normal (G1) or MET (G3) groups.

**Table 2:** Hypoglycemic effects of *S. officinalis* AE and EE extracts on RBG and FBG in STZ-induced type 2 diabetes and oxidative stress in experimental rats (mean ± SE), n = 7.

Groups	RBG (mg/dL)				FBG (mg/dL)

	Weak-0	Weak-2	Weak-4	Weak-6	
NR	111.71 ± 1.58^d^	110.71 ± 2.78^c^	108.71 ± 1.96^c^	113.29 ± 1.32^e^	87.02 ± 4.19^c^
STZ	311.57 ± 16.08^c^	319.14 ± 16.12^b^	329.57 ± 9.51^a^	346.86 ± 8.08^a^	176.92 ± 11.38^a^
MET	321.86 ± 17.21^bc^	298.71 ± 13.79^b^	279.86 ± 12.07^a^	276.71 ± 12.15^b^	89.96 ± 2.50^c^
STZ + AE400	353.43 ± 30.46^b^	323.43 ± 10.43^a^	290.86 ± 29.07^a^	216.00 ± 30.99^c^	122.80 ± 11.65^b^
STZ + AE800	390.57 ± 26.31^a^	303.71 ± 17.99^b^	238.57 ± 19.45^b^	172.71 ± 19.61^cd^	91.00 ± 8.09^c^
STZ + EE400	418.43 ± 17.39^a^	318.43 ± 16.15^b^	220.00 ± 11.86^b^	160.29 ± 18.65^d^	105.15 ± 8.70^bc^
STZ + EE800	391.43 ± 20.98^a^	354.86 ± 9.26^a^	204.00 ± 8.21^b^	146.14 ± 8.41^de^	84.89 ± 10.53^c^

G1=The negative group, G2=The positive group, G3=Diabetic rats + 50 mg Metformin/kg daily, G4=Diabetic rats + 400 mg of *S. officinalis* AE/kg daily, G4=Diabetic rats + 800 mg of *S. officinalis* AE/kg daily, G6=Diabetic rats + 400 mg of *S. officinalis* EE/kg daily; and G7=Diabetic rats + 800 mg of *S. officinalis* EE/kg daily, RBG=Random blood glucose, FBG=Fasting blood glucose level measured in blood serum of 12-h fasted rats, ^a,b,c^There is no significant difference (p > 0.05) between any two means, within the same column have the same superscripted letters. *S. officinalis*=*Salvia officinalis*, AE=Aqueous, EE=Ethanolic, STZ=Streptozotocin, MET=Metformin, SE=Standard error, NR=Normal rats

### Hypolipidemic efficiency of the AE and EE extracts of *S. officinalis* leaves

Table 3 shows the hypolipidemic efficacy of AE and EE of *S. officinalis* leaves at 400, 800 mg/kg, and MET at 50 mg/kg on STZ-induced diabetic rats. In diabetic rats, TG, CHO, LDL, and VLDL levels were increased significantly. However, STZ administration markedly declined HDL levels compared with healthy rats (G1). Compared with the normal rats (G1) and MET groups (G3), the TG, CHO, LDL-cholesterol (LDL-C), and VLDL-C levels of the rats treated with AE and EE of *S. officinalis* leaves at 400 and 800 mg/kg were considerably attenuated. The AE and EE of *S. officinalis* leaves at 800 mg/kg were the most effective treatments for enhancing the blood profile; even the EE of *S. officinalis* leaves was considerably better than the AE of *S. officinalis* leaves. TG levels were attenuated by 31.45, 44.34, 35.97, and 42.82% when rats were treated with AE and EE of *S. officinalis* leaves at 400 and 800 mg/kg, respectively. Enchantingly, the HDL-cholesterol (HDL-C) increased rates were 51.05 and 48.92% for AE and EE at 800 mg/kg, respectively. In contrast, the decrease in LDL-C levels was 56.72% and 67.94% after the administration of AE and EE at 800 mg/kg, respectively. The VLDL-C levels improved in parallel with treatments in a type- and dose-dependent manner. Compared with the STZ-group (G2), the VLDL decreased by 44.34% and 42.81% when administered 800 mg/kg of AE and EE of *S. officinalis* leaves.

**Table 3:** Effect of the AE and EE extracts of *S. officinalis* L. on the lipid profile in streptozotocin-induced type 2 diabetes and oxidative stress in experimental rats (mean ± SE), n = 7.

Groups	Lipid profile parameters (mg/dL)				

	TG	CHO	HDL-C	LDL-C	VLDL-C
NR	75.42 ± 3.21^d^	92.40 ± 6.73^d^	45.68 ± 3.68^a^	31.64 ± 9.70^b^	15.08 ± 0.64^d^
STZ	147.14 ± 7.08^a^	155.95 ± 12.37^a^	32.05 ± 4.88^c^	94.48 ± 15.82^a^	29.43 ± 1.42^a^
MET	97.39 ± 8.71^bc^	103.18 ± 7.62^bc^	43.64 ± 4.05^a^	40.07 ± 8.13^b^	19.48 ± 1.74^bc^
STZ + AE400	100.86 ± 5.62^b^	114.64 ± 6.92^bc^	40.23 ± 5.75^ab^	54.24 ± 8.72^b^	20.17 ± 1.12^b^
STZ + AE800	81.90 ± 3.95^cd^	104.93 ± 6.65^b^	48.41 ± 4.22^a^	40.89 ± 9.57^b^	16.38 ± 0.79^cd^
STZ + EE400	94.22 ± 6.7^bc^	103.18 ± 2.8^bcd^	41.59 ± 3.12^ab^	42.75 ± 4.54^b^	18.84 ± 1.34^bc^
STZ + EE800	84.14 ± 5.94^bcd^	93.60 ± 5.26^cd^	47.73 ± 2.46^a^	30.29 ± 4.37^b^	16.83 ± 1.19^bcd^

G1=The negative group, G2=The positive group, G3=Diabetic rats + 50 mg Metformin/kg daily, G4=Diabetic rats + 400 mg of *S. officinalis* AE/kg daily, G4=Diabetic rats + 800 mg of *S. officinalis* AE/kg daily, G6=Diabetic rats + 400 mg of *S. officinalis* EE/kg daily, and G7=Diabetic rats + 800 mg of *S. officinalis* EE/kg daily, TG=Triglycerides, CHO=Total cholesterols, HDL-C=High-density lipoprotein-cholesterols, LDL-C=Low-density lipoprotein-cholesterols, VLDL-C=Very low-density lipoprotein-cholesterols, ^a,b,c,d^There is no significant difference (p > 0.05) between any two means within the same column with the same superscripted letters. *S. officinalis*=*Salvia officinalis*, AE=Aqueous, EE=Ethanolic, STZ=Streptozotocin, MET=Metformin, SE=Standard error, NR=Normal rats

### Effects of the AE and EE extracts of *S. officinalis* leaves on the AI

**Figure 1:**
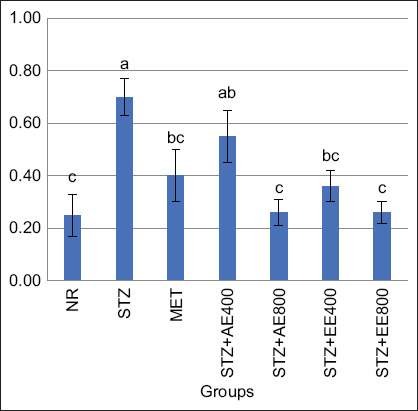
Effect of aqueous and ethanolic extracts of *Salvia officinalis* L. on AI in streptozotocin-induced type 2 diabetes and oxidative stress in experimental rats (mean ± SE), G1=The negative group, G2=The positive group, G3=Diabetic rats + 50 mg Metformin/kg daily, G4=Diabetic rats + 400 mg of *S. officinalis* AE/kg daily, G5=Diabetic rats + 800 mg of *S. officinalis* AE/kg daily, G6=Diabetic rats + 400 mg of *S. officinalis* EE/kg daily, and G7=Diabetic rats + 800 mg of *S. officinalis* EE/kg daily, n = 7. *^a,b,c^*Bars not sharing similar letters differed significantly (p > 0.05).

The efficiency of AE and EE of *S. officinalis* leaves at 400, 800 mg/kg, and MET at 50 mg/kg on AI in STZ-induced diabetes in rats were calculated; data are illustrated in Figure 1. Interestingly, STZ injection (G2) significantly boosted AI compared with the control (G1). The most effective treatments for attenuating atherogenicity complications were obtained after using AE and EE of *S. officinalis* leave at 800 mg/kg (G5 and G7), which present an enhanced effect that is better than AE and EE of *S. officinalis* leave at 400 mg/kg (G4 and G6), or even with MET (G3).

### Effects of the AE and EE extracts of *S. officinalis* leaves on liver function.

Table 4 shows the effects of AE and EE of *S. officinalis* leaves at 400, 800 mg/kg, and MET at 50 mg/kg on liver function in rats with STZ-induced diabetes. STZ injection significantly increased blood ALT, AST, and ALP enzyme levels in G2 rats with diabetes complications compared with normal rats (GI). STZ-treated rats had considerably elevated T. Bili levels (Table 4). Administrating the AE and EE of *S. officinalis* leaves at 400 and 800 mg/kg improved the liver’s function in a dose-dependent manner. Compared with lower doses, higher doses of AE and EE from *S. officinalis* leaves were more effective at restoring normal hepatic metabolism after STZ injection (Table 4). In the same context, a comparison of a lower dose at 400 mg/kg of AE or EE with 800 mg/kg exhibited that the administration of 400 mg/kg attenuated ALT by 43.12% and 42.23% against 48.44 and 46.12%, respectively. A similar trend was observed for AST, whereas 16.84 and 24.31 were recorded for AE of *S. officinalis* at 400 and 800 mg/kg.

**Table 4:** Effects of the AE and EE extracts of *S. officinalis* L. on liver function in STZ-induced type 2 diabetes and oxidative stress in experimental rats (mean ± SE), n = 7.

Groups	Liver’s functions					

	ALT (U/L)	AST (U/L)		ALP (U/L)		Total bilirubin (mg/dL)
NR	74.78 ± 27.77^b^	96.19 ± 5.58^b^		99.06 ± 2.68^bc^		0.59 ± 0.15^b^
STZ	149.05 ± 20.83^a^	147.9 ± 25.77^a^		164.15 ± 6.75^a^		0.97 ± 0.18^a^
MET	101.06 ± 10.58^b^	103.25 ± 3.73^b^		102.81 ± 10.15^bc^		0.79 ± 0.08^ab^
STZ + AE400	84.78 ± 11.91^b^	122.99 ± 12.85^ab^		108.22 ± 7.34^b^		0.89 ± 0.16^ab^
STZ + AE800	76.85 ± 7.74^b^	111.95 ± 8.63^ab^		92.44 ± 7.33^bc^		0.65 ± 0.08^ab^
STZ + EE400	86.07 ± 12.25^b^	114.75 ± 13.63^ab^		104.47 ± 8.72^bc^		0.70 ± 0.05^ab^
STZ + EE800	80.33 ± 3.97^b^	103.2 ± 10.41^b^		84.72 ± 9.20^c^		0.57 ± 0.02^b^

	**Total protein (g/dL)**		**Albumin (g/dL)**		**Globulin (g/dL)**	

NR	8.89 ± 0.17^b^		4.32 ± 0.25^b^		4.58 ± 0.19^a^	
STZ	7.27 ± 0.29^d^		3.53 ± 0.08^c^		3.75 ± 0.31^a^	
MET	7.87 ± 0.36^cd^		4.01 ± 0.31^b^		3.86 ± 0.57^a^	
STZ + AE400	7.37 ± 0.21		3.69 ± 0.20^bc^		3.68 ± 0.34^a^	
STZ + AE800	8.31 ± 0.41^bc^		3.73 ± 0.29^bc^		4.58 ± 0.51^a^	
STZ + EE400	8.13 ± 0.46^bcd^		3.77 ± 0.27^bc^		4.36 ± 0.65^a^	
STZ + EE800	10.03 ± 0.33^a^		5.21 ± 0.37^a^		4.83 ± 0.53^a^	

G1=The negative group, G2=The positive group, G3=Diabetic rats + 50 mg Metformin/kg daily, G4=Diabetic rats + 400 mg of *S. officinalis* AE/kg daily, G4=Diabetic rats + 800 mg of *S. officinalis* AE/kg daily, G6=Diabetic rats + 400 mg of *S. officinalis* EE/kg daily, and G7=Diabetic rats + 800 mg of *S. officinalis* EE/kg daily, ALT=Alanine aminotransferase, AST=Aspartate aminotransferase, ALP=Alkaline phosphatase, T. Bili=Total bilirubin, ^a,b,c,d^No significant difference (p > 0.05) between any two means within the same column have the same superscripted letters. *S. officinalis*=*Salvia officinalis*, AE=Aqueous, EE=Ethanolic, STZ=Streptozotocin, MET=Metformin, SE=Standard error, NR=Normal rats

In comparison, 24.30 and 30.22% were recorded for EE of *S. officinalis* at 400 and 800 mg/kg, respectively. Higher efficiency was noticed for the AE and EE of *S. officinalis* at 400 and 800 mg/kg on ALP. Improvements of 34.07% and 43.69% for AE and 36.36% and 48.39% for EE at 400 and 800 mg/kg were observed, respectively. The best treatment was EE of *S. officinalis* at 800 mg/kg, which improved liver enzymes (ALT, AST, and ALP), and liver functions such as T. Bili were better than MET in a type- and dose-dependent manner. In the same table, compared to normal rats (GI), STZ-treated rats had considerably lower T. protein, albumin, and globulin levels. High doses of AE and EE of *S. officinalis* significantly attenuated changes in T. protein, albumin, and globulin levels to near or above-average levels in the GI tract. Compared with NR, *S. officinalis* EE at 800 mg/kg showed the most remarkable improvement, even better than MET.

### Effects of the AE and EE extracts of *S. officinalis* leaves on kidney function

Table 5 shows the nephroprotective effects of *S. officinalis* leaves at 400, 800 mg/kg, and MET at 50 mg/kg in STZ-induced diabetic rats. Compared with normal rats (GI), STZ injection significantly elevated the blood creatinine, urea, and BUN levels of G2 rats. High doses of AE and EE in *S. officinalis* significantly reduced the changes in creatinine, urea, and BUN caused by diabetes complications. Compared with NR, *S. officinalis* EE at 800 mg/kg showed the most significant improvement, even better than MET. Creatinine, urea, and BUN levels were attenuated after the administration of AE and EE of *S. officinalis* at 400 and 800 mg/kg in a dose-dependent manner. Comparing a lower dose at 400 mg/kg of AE or EE with 800 mg/kg exhibited that administrating both extracts attenuated creatinine by 30.08 and 33.08% against 39.10 and 35.34% compared with the STZ group, respectively. A similar trend was observed in the urea level, whereas 37.61 and 42.18 were recorded for AE of *S. officinalis* at 400 and 800 mg/kg. In comparison, 34.78% and 47.31% were recorded for EE of *S. officinalis* at 400 and 800 mg/kg, respectively. Higher efficiency was noticed for the AE and EE of *S. officinalis* at 400 and 800 mg/kg on BUN. An improvement of 37.62, 42.18, 34.77, and 47.29% for AE and EE at both concentrations was noticed, respectively.

**Table 5:** Effect of the AE and EE extracts of *S. officinalis* L. on kidney function in STZ-induced type 2 diabetes and oxidative stress in experimental rats (mean ± SE), n = 7.

Group	Kidneys’ functions		

	Creatinine (mg/dL)	Urea (mg/dL)	BUN (mg/dL)
NR	0.73 ± 0.02^c^	34.18 ± 6.74^b^	16.07 ± 3.17^b^
STZ	1.33 ± 0.02^a^	68.66 ± 10.51^a^	32.27 ± 4.94^a^
MET	0.96 ± 0.07^b^	47.31 ± 6.04^b^	22.24 ± 2.84^b^
STZ + AE400	0.93 ± 0.10^b^	42.84 ± 2.34^b^	20.13 ± 1.10^b^
STZ + AE800	0.81 ± 0.07^bc^	39.70 ± 6.86^b^	18.66 ± 3.22^b^
STZ + EE400	0.89 ± 0.05^bc^	44.78 ± 11.26^b^	21.05 ± 5.29^b^
STZ + EE800	0.86 ± 0.05^bc^	36.18 ± 3.99^b^	17.01 ± 1.88^b^

G1=Negative group, G2=Positive group, G3=Diabetic rats + 50 mg Metformin/kg daily, G4=Diabetic rats + 400 mg of *S. officinalis* AE/kg daily, G4=Diabetic rats + 800 mg of *S. officinalis* AE/kg daily, G6=Diabetic rats + 400 mg of *S. officinalis* EE/kg daily, and G7=Diabetic rats + 800 mg of *S. officinalis* EE/kg daily, ^a,b,c^ No significant difference (p > 0.05) between any two means within the same column with the same superscripted letters. *S. officinalis*=*Salvia officinalis*, AE=Aqueous, EE=Ethanolic, STZ=Streptozotocin, MET=Metformin, SE=Standard error, NR=Normal rats, BUN=Blood urea nitrogen

### Effects of the AE and EE extracts of *S. officinalis* leaves on antioxidant biomarkers

As shown in Table 6, STZ injection dramatically reduced GSH, CAT, and SOD enzyme levels and elevated MDA levels in the blood serum of DR (G2) rats compared with normal rats (G1). Antioxidant enzyme activity (GSH, CAT, and SOD) and malondialdehyde (MDA) levels were significantly enhanced in rats given *S. officinalis* leaves at 400 and 800 mg/kg and MET at 50 mg/kg (Table 6). In comparison with the STZ-group (G2), GSH, MDA, CAT, and SOD improved by 75.02, 35.39, 42.10, and 59.64%, respectively, after treatment with AE of *S. officinalis* leaves at 800 mg/kg. Compared with the STZ group, the GSH, DMA, CAT, and SOD levels in those given 800 mg/kg of EE of *S. officinalis* leaves showed significant increases of 112.68, 39.77, 55.14, and 72.35%, respectively (G2). The enzymatic defense system was also significantly improved by MET compared to DR rats (G2).

**Table 6:** Effects of the AE and EE extracts of *S. officinalis* L. on antioxidant biomarkers in STZ-induced type 2 diabetes and oxidative stress in experimental rats (mean ± SE), n = 7.

Groups	Antioxidant biomarkers			

	GSH (μg/dL)	MDA (nmol/mL)	CAT (U/L)	SOD (U/L)
NR	82.60 ± 9.47^ab^	16.09 ± 0.55^d^	101.10 ± 7.50^abc^	99.80 ± 6.41^a^
STZ	41.71 ± 4.87^d^	26.48 ± 0.48^a^	73.21 ± 13.33^d^	53.45 ± 3.78^d^
MET	56.31 ± 5.30^cd^	19.22 ± 0.69^b^	83.45 ± 9.29^bcd^	66.68 ± 4.80^cd^
STZ + AE400	67.5 ± 10.41^bc^	18.81 ± 0.68^b^	77.73 ± 4.79^cd^	70.56 ± 6.12^bc^
STZ + AE800	73.00 ± 2.19^abc^	17.11 ± 0.33^cd^	104.03 ± 6.10^ab^	85.33 ± 6.84^ab^
STZ + EE400	60.47 ± 2.59^c^	18.15 ± 0.32^bc^	81.06 ± 10.10^bcd^	73.18 ± 4.47^bc^
STZ + EE800	88.71 ± 4.69^a^	15.95 ± 0.34^d^	113.58 ± 7.11^a^	92.12 ± 4.35^a^

G1=Negative group, G2=Positive group, G3=Diabetic rats + 50 mg Metformin/kg daily, G4=Diabetic rats + 400 mg of *S. officinalis* AE/kg daily, G4=Diabetic rats + 800 mg of *S. officinalis* AE/kg daily, G6=Diabetic rats + 400 mg of *S. officinalis* EE/kg daily, and G7=Diabetic rats + 800 mg of *S. officinalis* EE/kg daily, ^a,b,c^No significant difference (p > 0.05) between any two means within the same column with the same superscripted letters. *S. officinalis*=*Salvia officinalis*, AE=Aqueous, EE=Ethanolic, STZ=Streptozotocin, MET=Metformin, SE=Standard error, NR=Normal rats, GSH=Glutathione, MDA=Malonaldehyde, SOD=Superoxide dismutase, CAT=Catalase

### Effects of the AE and EE extracts of *S. officinalis* leaves on pancreatic histoarchitecture

**Figure 2:**
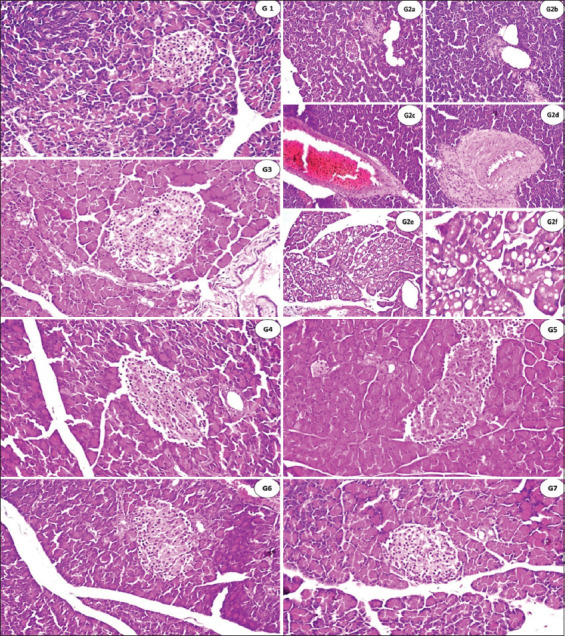
(G1) There were no histopathological alterations, and the normal histological structure of the islets of Langerhans as an endocrine portion as well as the acini and ducts system as an exocrine one was recorded. (G2a) Atrophy and regression in size as well as the shape of the islet of Langerhans cells, (G2b) cystic dilatation of the duct, (G2c) severe congestion in stromal blood vessels between the lobules, (G2d) sclerosis in the wall of congested stromal blood vessels, (G2e) fatty change in the lining epithelium of some acini in the lobules, and (G2f) magnification of this section to identify the fatty change in epithelium cells was shown. (G3) There was no histopathological alteration in the islet of Langerhans cells, while the ducts showed cystic dilatation. (G4, G5, G6, and G7) There was no histopathological alteration, as the normal size and shape of the islets of Langerhans cells were recorded.

The findings of histological examination corroborated those of biochemical studies. Histological changes in the pancreatic tissue of experimental groups of rats given *S. officinalis* leaves at 400, 800 mg/kg, and MET at 50 mg/kg are displayed in Table 7 and Figure 2. The pancreas of the control group showed no histopathological changes and a normal histological structure of both the endocrine (islets of Langerhans cells) and exocrine (the acini and ducts) (Figure 2G1). The histoarchitecture of the STZ-treated rats (Figure 2G2) showed atrophy, regression in size as well as shape, or complete absence of the islet of Langerhans cells noticed in most of the lobules (Figure 2G2a), cystic dilatation of the duct was shown in (Figure 2G2b), associated with congestion in the stromal blood vessels as shown in (Figure 2G2c), as well as sclerosis the vascular wall (Figure 2G2d). The fatty change was detected in the lining epithelium of some acini in a focal manner (Figure 2G2e), and the magnification of this section to identify the fatty change in epithelium cells is shown (G2f). In G3, there was no histopathological alteration in the islet of Langerhans cells, but only the duct changes showed cystic dilatation (Figure 2G3). No histological changes were found in the islets of Langerhans cells or the acini after the administration of 400 mg/kg and 800 mg/kg of *S. officinalis* leaves (Figure 2G4, G5, G6, and G7).

**Table 7:** Severity of histopathological alterations in the rat pancreas underlying the structure of different experimental groups treated with AE and EE at 400 and 800 mg/kg of *S. officinalis* L. (n = 3).

Alterations	Groups						

	NR	STZ	MET	STZ+AE400	STZ+AE800	STZ+EE400	STZ+EE400
Atrophy in Langerhans islets	–	+++	–	–	–	–	–
Necrobiosis of acini leaves	–	++	+	–	–	–	–
Interlobular fibrosis	–	++	+	+	–	–	–
Fatty tissue accumulation	–	+++	+	–	–	–	–

G1=The negative group, G2=The positive group, G3=Diabetic rats + 50 mg Metformin/kg daily, G4=Diabetic rats + 400 mg of *S. officinalis* AE/kg daily, G4=Diabetic rats + 800 mg of *S. officinalis* AE/kg daily, G6=Diabetic rats + 400 mg of *S. officinalis* EE/kg daily, and G7=Diabetic rats + 800 mg of *S. officinalis* EE/kg daily, +++=Severe, ++=Moderate, +=Mild, –=Nil, *S. officinalis*=*Salvia officinalis*, AE=Aqueous, EE=Ethanolic, STZ=Streptozotocin, MET=Metformin, NR=Normal rats

## DISCUSSION

Several herbs effectively manage diabetes through mechanisms including lowering blood pressure, increasing the synthesis of antioxidant enzymes, influencing the composition of gut bacteria, and preventing the excessive release of inflammatory cytokines [17, 20, 21, 51–53]. Sage is rich in phytochemicals and is often regarded as a powerful antioxidant because it neutralizes free radicals [54–56]. *S. officinalis* has been proven effective in recent trials as an antidiabetic and in reducing oxidative stress [17, 20, 21, 51]. Most diabetes problems are caused by hyperglycemia that lasts too long. Indeed, metabolic deficits and oxidative stress are thought to result from diabetes patients’ prolonged exposure to hyperglycemia [57]. Both AE and EE of *S. officinalis* significantly reduced RBG and FBG in experimental rats in a dose- and type-dependent manner, as established in our recent *in vivo* investigation [58, 59]. These results corroborate our study findings that *S. officinalis* has hypoglycemic properties. The polyphenols in *S. officinalis*, which are potent antioxidants, may contribute to the plant’s potential MET-like effect, which could be advantageous in preventing diabetes [60, 61]. *S. officinalis* AE and EE were most beneficial for restoring body weight in a dose- and type-dependent manner [62]. Therefore, the restoration of body weight observed in the diabetic animals in this study may be partly due to the ability of *S. officinalis* to attenuate hyperglycemia [29].

Impaired fat metabolism is linked to diabetic complications, as shown by elevated blood triglyceride and cholesterol levels in STZ-diabetes rats; histopathological analysis corroborated the first diagnosis. The AE and EE of *S. officinalis* at 400 and 800 mg/kg mitigated the extreme changes in lipid profiles compared with individual doses [60]. High doses of *S. officinalis* AE and EE exerted cumulative or synergistic effects by effectively lowering TG, CHO, LDL, and VLDL. Anti-oxidants, phenols, and carotenoids may be the leading causes of these activities [14]. Clinical investigations have shown that *S. officinalis* ingestion can reduce cholesterol levels and enhance the lipid profile, thereby aiding insulin regulation and reducing diabetic complications [63]. Impaired fat metabolism is linked to diabetic complications, as shown by elevated blood triglyceride and cholesterol levels in STZ-diabetes rats [64]; a histopathological analysis corroborated the first diagnosis. The AE and EE of *S. officinalis* at 400 and 800 mg/kg mitigated the extreme changes in lipid profiles compared with individual doses [60]. High doses of *S. officinalis* AE and EE exerted cumulative or synergistic effects by effectively lowering TG, CHO, LDL, and VLDL. Anti-oxidants, phenols, and carotenoids may be the leading causes of these activities [14]. Clinical investigations have shown that *S. officinalis* ingestion can reduce cholesterol levels and enhance the lipid profile, thereby aiding insulin regulation and reducing diabetic complications [63, 65]. Liver diagnostic indicators were significantly different between the treated and control groups (G1). In G2, ALT, AST, ALP, T. Bili, and D. Bili increase significantly in response to STZ treatment [66] as a normal deterioration related to liver injury in DM [67]. These increases were markedly reduced by MET, AE, and EE of *S. officinalis* at 400 and 800 mg/kg, respectively, in groups 3, 4, 5, 6, and 7, with the most significant reductions occurring in groups 5 and 7. Extracts of *S. officinalis* have been characterized as both anti-inflammatory and non-toxic to the liver, and their high phenolic acid concentration lends credence to these claims [14, 68].

The blood glucose levels in the STZ-injected rats (G2) increased dramatically, and the rats in this group also experienced a decline in kidney function. An increase in blood sugar also causes damage to the kidney filtering mechanisms, eventually resulting in renal failure; hence, the two conditions are tightly linked [69]. Consequently, DM has become a prominent cause of end-stage kidney disease, also known as diabetic nephropathy [70]. In contrast, oral administration of AE and EE of *S. officinalis* at 400 and 800 mg/kg resulted in a dose-dependent recovery of all kidney functions in diabetic rats. Compared with the other groups, including the negative control group (G1) and the group (G3) with MET treatment, G7 showed highly significant increases in T. protein, albumin, and globulin and decreases in creatinine, urea, and BUN. The antioxidant and free radical protection afforded by carnosic acid, rosmarinic acid, caffeic acid, and essential oil in *S. officinalis* extract contributes to its beneficial effect on restoring kidney function. Alternatively, we hypothesized that this extract has hypoglycemic effects in normal and diabetic animals. It decreases glucose synthesis in the liver and increases insulin action, which is associated with enhanced renal function [69].

Well-documented previous research has shown that STZ treatment significantly reduces GSH, SOD, and CAT levels and increases MDA levels in the serum of diabetic rats relative to normal rats. A non-enzymatic antioxidant called GSH is present in all mammalian cells. The oxidized form of GSH (Reduced glutathione) protects cells from oxidative stress by functioning as a cofactor for several different detoxifying enzymes (including GSH peroxidase, GSH S-transferase, and others) [71]. Similarly, SOD catalyzes the dismutation of two molecules of superoxide anion (*O_2_) into hydrogen peroxide (H_2_O_2_) and molecular oxygen (O_2_), reducing the toxicity of the original superoxide anion [72]. MDA is the primary product of lipid peroxidation and a potent indicator of oxidative stress. As measured by the catabolite malondialdehyde, ROS increases the risk of tissue damage and induces lipid peroxidation [73]. Treatment with MET (50 mg/kg) and AE and EE of *S. officinalis* (400 and 800 mg/kg) reduced STZ’s adverse side effects by reversing the decreased antioxidant enzyme activity of SOD, CAT, and GSH. In other cases, this may even shut down the mechanism responsible for MDA production [62]. Since it has been established that *S. officinalis* has a MET-like action, its AE and EE showed improved performance in anti-oxidation prevention compared to MET [23]. According to the results of the current investigation, lipid peroxidation was reduced, and antioxidant enzyme SOD and CAT levels were increased in STZ-diabetic rats after the oral administration of AE and EE of *S. officinalis* [62]; the efficiency was markedly increased in high doses of both EE of *S. officinalis*, presenting small molecules of “metformin-like” substances in addition to rich phenolic content that can modulate diabetes and attenuate its complications [60, 61]. *S. officinalis* AE and EE reduced the elevation in MDA levels and restored the overall antioxidant power in STZ-treated rats. *S. officinalis*, which has potent antioxidative activity in the presence of abundant polyphenols, effectively alleviates problems associated with oxidative stress [74].

The pancreas histoarchitecture under investigation in this study had typical histological structures, as seen in photomicrographs of the G1 segment. Normal, fully formed islets of Langerhans (a and b) are noticeably smaller and more irregular in shape in G2 (relative to G1) development. As a positive control among the study groups, the STZ-treated rats (50 mg/kg, i.p.), histoarchitecture showed atrophy, regression in size as well as shape, or complete absence of the islet of Langerhans cells in most of the lobules, cystic dilatation of the duct associated with congestion in the stromal blood vessels, as well as sclerosis of the vascular wall in addition to a fatty change in the lining epithelium of some acini in a focal manner, as shown in Figure 2G2a_f. This suggests that STZ is highly toxic to pancreatic cells, causing extensive damage to β-cells [75]. A comprehensive reduction in β-cell mass and destruction of pancreatic islet volume have previously been demonstrated by STZ in animal studies [76]. Moreover, the findings of El-Sheikh *et al*. [77] stated that the pancreatic cells of diabetic rats exhibited substantial damage and loss of architecture, including marked atrophy of the islets of Langerhans and a decrease in the number and size of β-cells. Abunasef *et al*. [78] and Alharbi *et al*. [17] provided previous explanations of the same finding and noted that STZ significantly diminished islet size and number, especially in the central region.

Histopathological examination of the islet of Langerhans cells in the pancreas of MET-treated rats (Figure 2G3) revealed no pathological changes. However, only cystic dilatation was the sole visible change in the ducts. Restoring Langerhans islet function is crucial in diabetes management, as was previously discussed by Mansour *et al*. [79], as targeting the pancreatic β-cells is considered one of the most promising strategies for treating diabetes [80]. *S. officinalis* extract improved STZ-induced diabetic rats (Figure 2G4-G7). When diabetic rats were administered AE and EE of *S. officinalis* at doses of 400 and 800 mg/kg, respectively, insulin secretion along with the activity of the Langerhans islets β-cells were restored. This may be because *S. officinalis* extracts are rich in phenolic and flavonoid compounds [19]. Researchers have found that *S. officinalis* extracts could treat various forms of diabetes because they prevent DNA damage, lower lipid peroxidation, shield brain cells from H_2_O_2_, and have hypoglycemic effects [81]. The pancreatic structural restoration was clearly visible in G4, G5, G6, and G7 animals (Figure 2G4, G5, G6 and G7). However, neither the concentration nor the type of *S. officinalis* extract demonstrated significant variation in pancreas histological alterations in the treated rats under the experiment. This could be explained by the fact that *S. officinalis* effectively attenuated the histological changes at the concentrations used in this study. For an accurate investigation of the antidiabetic potential of *S. officinalis*, collected histopathological and biochemical examination data should be combined and discussed in parallel. As previously highlighted, the antidiabetic potential of *S. officinalis* was confirmed [20, 21, 51] and agreed with our results; therefore, incorporating it into diet or feed is highly recommended.

## CONCLUSION

This study demonstrated that AE and EE extracts of *S. officinalis* L. leaves possess potent antidiabetic, hypolipidemic, and antioxidative properties in STZ-induced type 2 diabetic rats. Both AE and EE, particularly at 800 mg/kg body weight, significantly reduced RBG, FBG, and TG while improving HDL-C levels. In addition, the extracts restored liver and kidney functions and enhanced antioxidant enzyme activity (GSH, CAT, and SOD) while reducing oxidative stress (MDA). Histopathological analysis confirmed the structural restoration of pancreatic islets and acinar cells, underscoring the therapeutic potential of *S. officinalis*.

The study comprehensively evaluated multiple parameters, including biochemical, antioxidant, and histological markers, providing robust evidence for the therapeutic efficacy of *S. officinalis*. The use of a well-designed animal model and comparison with a standard treatment (Metformin) strengthens the reliability of the results. Furthermore, the dose-dependent effects of AE and EE highlight their potential for flexible therapeutic applications.

However, the study was limited to an animal model, and the results may not directly translate to humans without further clinical validation. In addition, the exact bioactive compounds responsible for the observed effects were not isolated or quantified, limiting the understanding of their mechanisms. The long-term effects and potential toxicity of high doses of *S. officinalis* were also not explored.

Further studies are needed to isolate and characterize the bioactive compounds in *S. officinalis* responsible for its antidiabetic and antioxidative effects. Clinical trials are essential to evaluate the efficacy, safety, and optimal dosage of *S. officinalis* extracts in human populations. Investigating the synergistic effects of *S. officinalis* with other antidiabetic therapies could pave the way for combination treatments. In addition, exploring the long-term safety and efficacy of *S. officinalis* in managing diabetes and preventing its complications is critical for its potential therapeutic use.

Overall, this study highlights the promise of *S. officinalis* as a natural therapeutic option for diabetes management and oxidative stress mitigation, laying the groundwork for future research and clinical applications.

## DATA AVAILABILITY

The data generated during the study are available within the article.

## AUTHORS’ CONTRIBUTIONS

HB and RAA: Conceptualization, methodology, investigation, and Writing – original draft preparation. HB and YMA: Data curation. HB and YMA: Review and editing. All authors have read and agreed to the publication of the final version of the manuscript.
